# Biochemical and Metabolomic Responses of Antarctic Bacterium *Planococcus* sp. O5 Induced by Copper Ion

**DOI:** 10.3390/toxics10060302

**Published:** 2022-06-02

**Authors:** Ziyi Cheng, Cuijuan Shi, Xiujun Gao, Xiaofei Wang, Guangfeng Kan

**Affiliations:** School of Marine Science and Technology, Harbin Institute of Technology, Huancui District, Weihai 264209, China; cheng1909901937@outlook.com (Z.C.); cjshi@hit.edu.cn (C.S.); sxlinlu@163.com (X.G.); wangxiaofei_hit@163.com (X.W.)

**Keywords:** Antarctic strain, copper stress, adaption responses, metabolomics

## Abstract

Heavy metal pollution in the Antarctic has gone beyond our imagination. Copper toxicity is a selective pressure on *Planococcus* sp. O5. We observed relatively broad tolerance in the polar bacterium. The heavy metal resistance pattern is Pb^2+^ > Cu^2+^ > Cd^2+^ > Hg^2+^ > Zn^2+^. In the study, we combined biochemical and metabolomics approaches to investigate the Cu^2+^ adaptation mechanisms of the Antarctic bacterium. Biochemical analysis revealed that copper treatment elevated the activity of antioxidants and enzymes, maintaining the bacterial redox state balance and normal cell division and growth. Metabolomics analysis demonstrated that fatty acids, amino acids, and carbohydrates played dominant roles in copper stress adaptation. The findings suggested that the adaptive mechanisms of strain O5 to copper stress included protein synthesis and repair, accumulation of organic permeable substances, up-regulation of energy metabolism, and the formation of fatty acids.

## 1. Introduction

Over the past few years, with the high level of industrial activities and widespread pesticides and fertilizers, heavy metals have been commonly detected in diverse environments around the world and gradually accumulated [1]. Furthermore, heavy metal concentrations can be subsequently biomagnified thousands of times through biological amplification in an ecosystem [2]. Therefore, these heavy metals seriously threaten the stability of the ecological system and the health of human beings [3].

Although metals play an essential role, directly or indirectly, in vital cellular processes such as aerobic metabolism and cellular respiration, heavy metal concentrations above the maximum threshold are toxic to living things by the alterations of nucleic acids and polypeptide conformation and the disturbance of cell wall integrity, enzyme specificity, oxidative phosphorylation, and osmotic balance [4,5]. As we all know, microorganisms are found almost everywhere on earth [6]. Microbes have activated defensive strategies and evolved several adaptation mechanisms for survival [7], such as accumulation on a cell wall, transportation across the cell membrane, a permeable membrane, intracellular sequestration, and enzymatic detoxifications [5,8]. The capability of organisms to modulate their metabolism is a central characteristic required for proliferation, hibernation, and survival [9]. The metabolic mechanism of microbial resistance to heavy metal could be elucidated in detail from different perspectives, which we shall describe next. Adjusting the fatty acid composition of the cell membrane and reconfiguring energy-generating processes have been seen as the most efficient adaptation mechanisms to heavy metals [10]. Under metal stress, partial microorganisms fulfill their energy requirements through substrate-level phosphorylation rather than oxidative phosphorylation [11]. For example, the resistance of *Proteobacteria* to heavy metals may be because the phylum can utilize a variety of organics as carbon and energy sources [12]. The presence of large amounts of long-chain fatty and phospholipid saturation contributes to low fluidity and rigidity of the membrane to prevent the bacterial cell from contaminants [13,14]. Active efflux and precipitation are in bacterial partial resistance toward metals [15], shown in the acidophilic bacterium *Acidithiobacillus ferrooxidans* and *Bacillus sphaericus* [16,17,18]. In *Escherichia coli*, the addition of copper (Cu) stimulates the degradation of intracellular polyphosphate, and phosphate exportation also supports this mechanism [19]. In addition, heavy metals are precipitated and eliminated by compounds produced by microorganisms under heavy metal stress, contributing to a degree of bacterial resistance to metals [15]. To circumvent aluminum (Al) toxicity, *Pseudomonas fluorescens* promotes the synthesis of citrate involved in the sequestration of Al [20]. *Desulfovibrio desulfuricans* can regulate the precipitation of metals by forming metal sulfides [21]. The metabolic level of low molecular weight organic acids can be up-regulated to dissolve heavy metals by using them as a final electron acceptor or decreasing pH [22,23]. *Oxalobacter formigens* absorb as minimal as possible Pb by converting it to oxalate that can be excreted from the gut [24]. Interestingly, bacteria increase stress response-related metabolites to rebalance oxidative stress and osmotic pressure damaged by heavy metals. For instance, *Scenedesmus obliquus* increases lipid esters and Cys-GSH isomers for antioxidant defense mechanisms and reactive oxygen species prevention under cadmium stress [25]. In addition, ethanol tolerance involves increased glycine metabolism, which serves as protective osmolytes in *Escherichia coli* [26].

The Antarctic, an isolated place and often considered a clean slate, is facing the challenge of negative factors derived from human activities [27,28,29]. Unfortunately, heavy metals have been detected in abiotic samples such as surface soil, atmosphere particulate, and snow in Antarctica [30]. Furthermore, heavy metal concentrations in Southern Ocean organisms are significantly higher than in other oceans [29]. The migration and accumulation of heavy metals have become one of the severe problems in Antarctica. In fact, Cu is one of the most common sources of heavy metals contributing to contamination in Antarctica [31]. As an essential micronutrient, Cu is employed by most organisms to perform different functions, such as acting as a catalytic cofactor in cellular redox reactions and metal homeostasis [32,33,34]. However, an excessive amount of copper can be toxic.

Although the adaptation strategies to Cu have been relatively well characterized, the metabolic reprogramming leading to stress-induced lifestyle changes in polar microorganisms remains a mystery. The sensitivity of organisms to contamination may vary with latitude [35]. Polar organisms have evolved unique characteristics to adapt to severe regimes at high latitudes, including lower metabolisms, longer lifespans, and higher lipid content in tissues [36]. However, most research focuses on low-temperature enzyme production and low-temperature adaptation mechanisms, which ignores unique metabolic mechanisms that adapt to the environment [37,38]. Strain O5 isolated from Antarctic sea ice was subsequently identified as *Planococcus* based on 16S rDNA sequence analysis [39].

In this study, cell growth and physiological and biochemical variations of the Antarctic bacterium *Planococcus* sp. O5 after Cu^2+^ exposure were analyzed to explore the tolerance mechanism of the bacterium to Cu. The results will help elucidate the adaptation mechanism of polar microorganisms under heavy metal exposure. Meanwhile, the strain has extensive tolerance to heavy metals, which can be applied to deal with the heavy metal pollution in Antarctic in the future.

## 2. Materials and Methods

### 2.1. Bacterial Strains and Culture

The Antarctic strain *Planococcus* sp. O5 was isolated from Antarctic sea ice collected by the 23rd China Antarctic scientific expedition. To investigate the growth effect of Cu^2+^ exposure, strain O5 was cultured in 2216E liquid medium (5.0 g of peptone, 1.0 g of yeast extract, and 0.015 g of iron phosphate tetrahydrate in 1000 mL of purified and sterilized seawater) at 10 °C with the agitation of 120 rpm. Additional CuSO4 (final concentration 0.5 mmol/L) was added as copper stress.

### 2.2. MIC Determination of Heavy Metals

The minimum inhibitory concentration (MIC) of strain O5 was tested as described by Rajpert [40]. Various metal resistance tests were performed in log phase culture of strains that were inoculated in 2216E liquid medium supplemented with Cu^2+^, Cd^2+^, Pb^2+^, Zn^2+^, and Hg^2+^ in the concentration ranging from 0 mM to 1500 mM. Strain growth was monitored using OD_595_ measurement with a UV spectrophotometer. The lowest metal concentration that hampered growth was regarded as the MIC of the test strain against metal.

### 2.3. Measurements of Electrical Conductivity and Biomass

The membrane permeability was detected using a conductivity meter, and the biomass was measured using OD_595_ [41].

### 2.4. Measurements of Antioxidant System

Changes in the antioxidant enzyme activities were determined to understand the influence of Antarctic bacterium under copper stress. For this purpose, 100 mg of fresh weight (FW) bacterial strain was homogenized in 20 mL 50 mM phosphate buffer (pH 7.8) using a prechilled mortar and pestle before centrifugation at 12,000 rpm for 30 min at 4 °C. The collected supernatants were employed to determine the antioxidant enzyme activities of superoxide dismutase (SOD), glutathione reductase (GR), ascorbate peroxidase (APX), and the content of glutathione (GSH) and carotenoid.

SOD activity was assayed as described by Zhang based on the inhibition of the oxidation inhibition rate of pyrogallol reaching 50% [42], and GR was measured following the method of Pinto, Mata, and Lopezbarea. GR activity was measured in OD_340_/(min•g FW) [43]. APX activity was measured using the method of Nakano, Y. and Asada, K. A unit of the enzyme activity was defined as ascorbic acid consumed by the bacterium (min•g FW) [44]. GSH content was performed as per the report described by Yoon and was measured in OD_400_ [45]. The multiparameter flow cytometry method documented by Freitas [46] was used to assay carotenoid content.

### 2.5. GC-MS analysis of Metabolites

#### 2.5.1. Sample Preparation

The bacteria supplemented with 0.5 mmol/L Cu^2+^ in the logarithmic and stable phases were acquired by centrifugation at 12,000 rpm at 4 °C for 5 min, respectively. Subsequently, 2 mL of 60% precooled methanol (−40 °C) were added and placed on ice for 5 min to quench the cellular reaction. After centrifugation, the collected cell pellet was introduced to 0.5 mL of methanol (50%, −40 °C), followed by rupturing with the sonication method. The broken cells were centrifuged (4 °C, 12,000 rpm, 10 min), and 10 μL of succinic-2,2,3,3-d 4 acid (0.3 mg/mL) was added to the supernatant. When the sample was dried, 100 μL of 20 mg/mL pyridine amine hydrochloride was added and subsequently oxidated at 30 °C for 1.5 h. Afterward, MSTFA (100 mL) was used to derivate the samples by incubation at 37 °C for 0.5 h.

#### 2.5.2. GC-MS Analysis of Metabolites

The metabolites were analyzed using GC-TOF-MS (Agilent 7890A, Santa Clara, CA, USA), and 1 μL of the derivatized sample was injected into GC-MS, which was equipped with a DB-FFAP capillary column (60m × 0.25 μm × 0.25μm). The elution program setting: isothermal at 80 °C for 1 min, then an increase of 2 °C min^−1^ up to 100 °C, and ramped at 4 °C min^−1^ to 240 °C, and then held for 15 min at 240 °C. The ion source temperature was maintained at 200 °C. The mass spectrometer was set to scan a mass range of 50–800 *m*/*z* at 20 scans/s with an electron beam of 70 eV.

#### 2.5.3. Data Processing

The peak integration and peak alignment were conducted by applying the XCMS package of R software, and components were manually identified and confirmed using the NIST library. Noise and low abundance components were eliminated from the data matrix based on a noise threshold (S/N > 10). The ultimate two-dimensional matrix consisted of retention time (RT) and mass-to-charge ratio (*m*/*z*) data pairs.

#### 2.5.4. Statistical Analysis

The processed data matrix was submitted to the MetaboAnalyst 4.0 (http://www.metaboanalyst.ca/, accessed on 4 March 2022) to conduct data pre-processing and multivariate statistical analysis. Data were normalized to total integral normalization before being log-transformed. Principal component analysis (PCA) was performed to provide a general overview and remove irrelevant variables. Orthogonal partial least squares discriminant analysis (OPLS-DA) was conducted to further investigate the metabolic variation. Metabolites of interest were filtered using volcano plots with fold change (FC) ≥1.2 and *p*-value < 0.05. To explore the related metabolic pathways for differential metabolites, compounds of interest were imported into the Pathway modules of MetaboAnalyst.

## 3. Results

### 3.1. Heavy Metals Resistance Analysis

**Figure 1:**
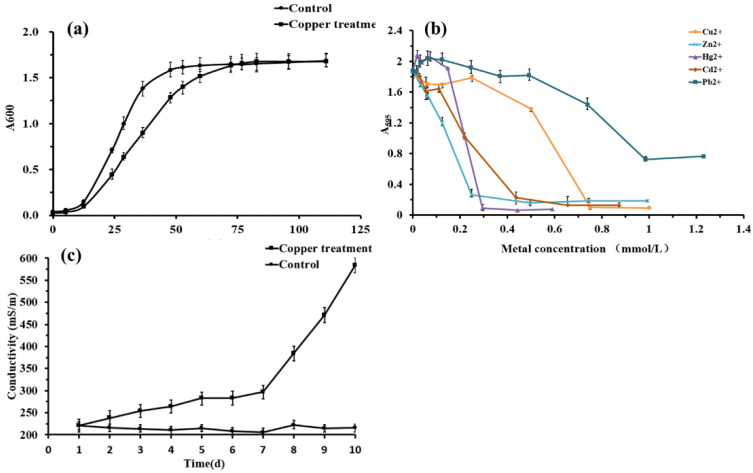
(**a**) Growth curves of the *Planococcus* sp. O5 in the absence and presence of 0.5 mmol/L Cu^2+^. (**b**) Heavy metal tolerance of *Planococcus* sp. O5 of Cu^2+^, Zn^2+^, Pb^2+^, Cd^2+^, and Hg^2+^, separately. (**c**) Effect of 0.5 mmol/L Cu^2^^+^ on the conductivity of *Planococcus* sp. O5.

Typical bacterial growth was observed in the induced and normal groups (Figure 1a). Although the growth rate of *Planococcus* sp. O5 in the normal group was higher in the induced group in the first 72 h, the growth of the copper exposure group presented an equal OD_595_ value to the untreated group subsequently. The relative metal resistance of strain O5 was in the order of Pb^2+^ > Cu^2+^ > Cd^2+^ > Hg^2+^ > Zn^2+^ (Figure 1b), and the MIC reached 1.0 mmol/L, 0.8 mmol/L, 0.7 mmol/L, 0.6 mmol/L, 0.5 mmol/L, respectively. Meanwhile, the optimum concentration of copper stress was determined to be 0.5 mmol/L.

### 3.2. Change in the Membrane Permeability

Electrical conductivity provides an indirect indication of membrane permeability. The conductivity of the control was stably maintained between 200–230 mS/m, as shown in Figure 1c. Correspondingly, the conductivity of the bacteria-induced with Cu^2+^ increased slowly on days 1–7 before dramatically increasing on day 10, reaching 586 mS/m. These findings suggested that the integrity of the cell membrane was altered after stimulation with 0.5 mmol/L Cu*^2+^*.

### 3.3. Response of the Antioxidant System

**Figure 2:**
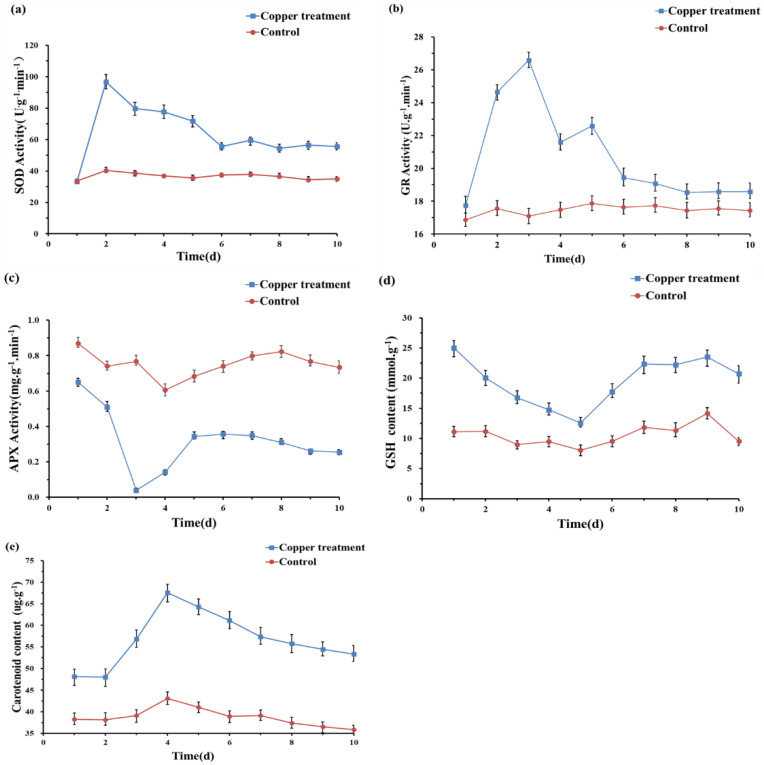
Effects of 0.5 mmol/L Cu^2+^ on the activities of SOD (**a**), GR (**b**), APX (**c**), GSH (**d**), and Carotenoid (**e**) of *Planococcus* sp. O5.

We also measured the changes in the content of antioxidant substances and the activities of antioxidant enzymes further to understand the biochemical mechanism of strain O5 to Cu, as shown in Figure 2. The enzymatic activity of SOD, GR, and APX remained almost unchanged throughout the experiment without Cu^2+^ induction. However, SOD and GR activities rose fast after the exposure to 0.5 mmol/L Cu^2+^, reaching their maximum value on day 2 and 3, respectively. In contrast, APX activity decreased and was significantly lower than the control. Carotenoid and GSH content accumulated rapidly under 0.5 mmol/L Cu^2+^ stress and remained considerably higher content than in the untreated group.

### 3.4. Metabolic Response of Strain O5 to Cu Induction

#### 3.4.1. Metabolic Profile Analysis

**Figure 3:**
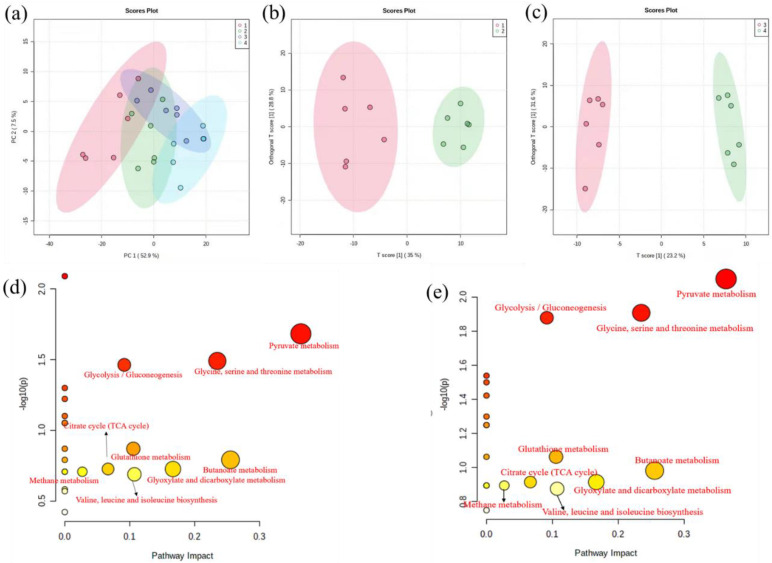
(**a**) shows the PCA models for 24 samples; numbers 1,2 represent the control group and copper exposure in the logarithmic phase, and numbers 3,4 represent the control group and copper exposure in the stationary growth phase. (**b**,**c**) separation of control (red) and copper exposure (green) samples in logarithmic phase and stationary growth phase using OPLS-DA, respectively. (**d**,**e**) metabolites changes mapped to the metabolic pathways exposed to 0.5 mmol/L Cu^2+^ of *Planococcus* sp. O5 in the logarithmic and stationary growth phase.

In the PCA score plot (Figure 3a), all 24 data samples were within the 95% confidence ellipse, indicating that no sample contained outliers. Although there was a noticeable separation between the Cu exposure and the control groups in the PCA, the treatment and control groups were intermixed. To further investigate the metabolic variation induced by copper stress, OPLS-DA was performed. As demonstrated in Figure 3b,c, the treatment and control groups showed a more distinct separation. The R2 values for the OPLS-DA model were satisfactory (>0.945, >0.99), explaining the majority of the variance between the samples. The Q2 values were much higher (>0.728, 0.7), indicating that the vast majority of the variation was to be expected.

#### 3.4.2. Identification and Analysis of Differential Metabolites

To discover the significant alterations of metabolites induced by copper exposure, we evaluated the changes in metabolites abundances using the filtering function of volcano plots. In total, 13 different metabolites were filtered based on the FC and the *p*-value with 4 significantly reduced and 9 increased (Table S1). The differential metabolites included energy, amino acid acids, and organic acids.

#### 3.4.3. Perturbed Biological Pathway Responded to Copper Stress

Pathway analysis was performed to investigate relevant pathways connected to Cu response, with the results shown in Figure 3d,e. The color shades and circle size were based on p-values and pathway impact values. Redder and large pathway circles indicated that the pathway was greatly perturbed. MetaboAnalyst Pathway found 7 key metabolic pathways in the logarithmic phase using pathway enrichment analysis, with pyruvate metabolism, butanoate metabolism, and glycine serine and threonine metabolism showing the most pronounced changes. Similarly, the most significant changes in the metabolic pathway also had the most critical impact during the stable growth phase. The *p*-value and impact factor of significant pathways are shown in Tables S2 and S3.

## 4. Discussion

Heavy metal ions, which are highly toxic, non-degradable, bioaccumulate, and biomagnifying as a result of the food chain, constitute a severe threat to ecological environments. Copper ions are micronutrients essential for the biological functions of living organisms. However, excess copper ions in cells are detrimental through various induced physiological, biochemical, and genotoxic effects [47]. Only copper-resistant microorganisms can strive and utilize trace metals to achieve metabolic functions while resisting or detoxifying their excesses. To discover the adaptation strategies of Antarctic microorganisms to heavy metal (Cu) stress, we combined biochemical and metabolomics methods to analyze the bacterium *Planococcus* sp. O5, isolated from the Antarctic sea ice sample.

### 4.1. Heavy Metals Resistance

In this study, the Antarctic bacterium *Planococcus* sp. O5 exhibited a relatively broad tolerance to Cu^2+^, Hg^2+^, Zn^2+^, Cd^2+^ and Pb^2+^, and especially Pb^2+^ and Cu^2+^ (Figure 1b). Similar bacterial resistance to multiple heavy metals was reported in other Antarctic strains, such as Antarctic *Rhodotorula mucilaginosa* resistance pattern of Cd^2+^ > Pb^2+^ = Mn^2+^ > Cu^2+^ > Cr^3+^ > Hg^2+^, and Antarctic bacteria isolated from rock lichen resistance pattern of Cr^3+^ > Ni^3+^ > Cu^2+^ > Co^2+^ > Hg^2+^ [48,49]. Multiple heavy metals resistance is attributed to these metals with similar toxic mechanisms and detoxifying processes [50]. In addition, the growth curve of bacteria appeared that the Antarctic sea-ice bacterium challenged by copper has adapted to the presence of 0.5 mmol/L Cu^2+^ (Figure 1a). In addition, the emergence of a lag phase may be due to oxidative stress induced by copper requiring the consumption of glutathione (GSH), whose synthesis requires additional energy. A similar adaptation mechanism has been observed in *Aspergillus niger* [51].

### 4.2. Effect on Redox Status

**Figure 4:**
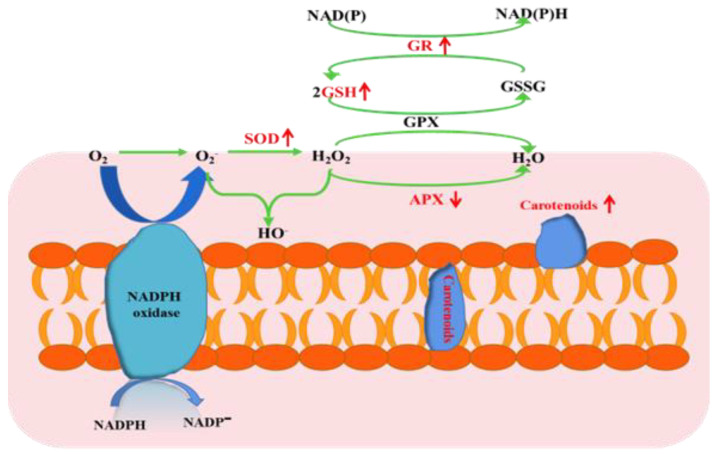
Biochemical reveals *Planococcus* sp. O5 copper tolerance mechanisms. The down and up arrow means down-regulation and up-regulation, respectively.

Reactive oxygen species (ROS), including superoxide anion (O^2−^), hydroxyl radical (∙OH), and hydrogen peroxide (H_2_O_2_), are among the significant toxicities of heavy metals to most living organisms by altering the reducing environment [52,53]. The membrane structure of polar bacterium was damaged by lipid peroxidation (Figure 1c), and similar responses occurred in other microorganisms [54]. However, strain O5 could take advantage of various antioxidative defense systems to rebalance the redox status (Figure 4) [55]. Glutathione (GSH), an essential indicator of the redox environment, plays a major role in cellular defense response against oxidative stress [56]. The copper resistance expressed in our study may also be explained by the activation of glutathione, which is rich in thiol groups and may be related to a metal complexation mechanism via a rich sulfur bond. This detoxification mechanism has been demonstrated in yeast [57]. Therefore, the increase in GR activity was entirely expected. The GR activity boosted the regeneration efficiency of GSH to maintain the intracellular redox balance. GR catalyzes GSSG into GSH in the presence of coenzyme *β*-nicotinamide adenine dinucleotide 2′-phosphate hydrate (NADPH) [58], which has been regarded as being related to resistance to oxidative stress in a microorganism [59]. Carotenoids are mainly located in the cell membrane, acting as antioxidant protectants for the cell membrane integrity [60]. In short, it is of great significance to regulate different antioxidant mechanisms when facing endogenous redox damage induced by copper.

### 4.3. Metabolic Reprogramming

**Figure 5:**
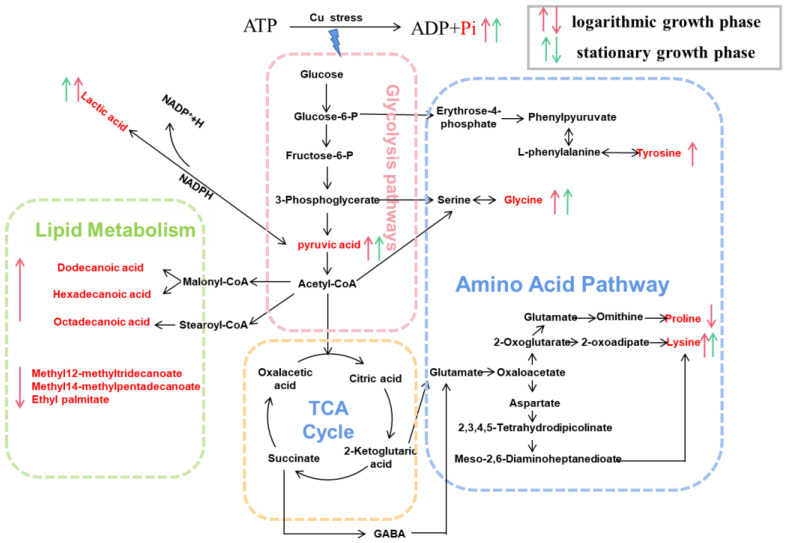
Metabonomics reveals *Planococcus* sp. O5 copper tolerance mechanisms. The identified significantly different metabolites were integrated into pathways. The down and up arrow means down-regulation and up-regulation, respectively.

The pathway enrichment analysis indicated that copper stress could generate metabolic reprogramming, resulting in alterations in many metabolites, particularly in energy, amino acid acids, and lipid metabolism, forming a metabolic network to deal with copper stress (Figure 5).

#### 4.3.1. Energy Metabolism

Carbohydrate and energy metabolism acted as key attributions in the adaptive responses to heavy metals [49]. In organisms, lactic acid is a byproduct of anaerobic metabolism, and pyruvic, an end product of glycolysis by the Embden Meyerhof pathway, is conversed to lactate acid by oxidizing nicotinamide adenine dinucleotide (NADH) in the presence of lactate dehydrogenase [61]. Bacteria activated the breakdown of sugars to generate more energy and amino acids as a defensive mechanism when exposed to copper. As a result, pyruvate acid and NADPH accumulated abundantly, leading to an aggregation of lactic acid. This energy model might alleviate the cytotoxicity of extreme Cu by reducing ROS levels [62].

#### 4.3.2. Amino Acid Metabolism

Amino acids are commonly used in all living cells for osmoregulation, energy sources, protein synthesis, metabolite precursors, and signaling molecules [63]. Glycine, an organic osmolyte [64], was reported to be closely associated with the Cu^2+^ tolerance of *Pseudomonas* [26]. In addition, glycine is a precursor of glutathione [65] and can alleviate oxidative damage. A previous study demonstrated that lysine altered NADPH flux to produce glutathione, enhancing tolerance to oxidative stress [66]. Hence, in this study, lysine-overproducing was expected to exhibit higher tolerance to copper stress. Surprisingly, proline content declined, which had not been observed in other organisms responding to environmental stresses [67,68,69,70]. Proline metabolism is highly relevant to redox homeostasis, protein and nucleotide synthesis, and ATP production, and is especially closely associated with the progression of oxidative stress [71]. Consequently, in this study, the down-regulation of proline demonstrated the oxidative balance was disrupted under copper stress, leading to lipid peroxidation [72]. Tyr is susceptible to modification under conditions of cellular redox imbalance. The oxidation of phenylalanine is a marker of oxidative stress [73]. Under conditions of ROS overaccumulation, tyrosine can be formed by phenylalanine hydroxylation or oxidation [74]. Increased concentrations of Tyr were also observed in plants in response to biological stress [75]. Moreover, the upregulation of glycine, lysine, and tyrosine was considered to repair damaged proteins and activate the synthesis of newer proteins [76]. The mentioned studies implicated the protein biosynthesis mechanisms enhanced by excessive copper [77].

#### 4.3.3. Lipid Metabolism

Stearic acid and palmitic acid were identified as quantitative markers of cellular stress, with which the overproduction and accumulation of ROS have been frequently associated [78]. This research implied the disruption of membrane integrity by lipid peroxidation (Figure 1c). This damage mechanism induced by copper has also been previously reported in filamentous fungus *Paecilomyces marquandii* [79]. Membranes of bacteria consist mainly of a lipid bilayer and embedded proteins, which allow solutes to selectively transport substances across the membrane to facilitate physiological processes such as respiration and signal transduction [80]. Integrity and fluidity of cell membranes influenced by lipids composition and the unsaturation degree of fatty acids are critical for organism survival in response to external stress [81]. Stress factors usually lead to lipid metabolism reconfiguration, resulting in decreased or increased membrane fluidity [82]. A low fluidity of the cell membrane can be perceived to effectively prevent copper ions from entering the bacteria cells [83,84]. As expected, saturated fatty acids have identified upregulation. In brief, the regulation of fatty acids significantly enhanced the resistance of copper and oxidative.

Copper stress resulted in metabolic reprogramming, according to metabolic profile analyses. In response to copper adaptation, bacteria activated the breakdown of intracellular sugars to generate more energy. In addition, amino acids support diverse functions, including maintaining appropriate cell status by increasing osmotic substances, producing new stress proteins, and repairing damaged or misfolded proteins. Furthermore, the accumulation of fatty acids may reduce the fluidity of the cell membrane, which can prevent copper ions from entering the bacteria cells.

## 5. Conclusions

*Planococcus* sp. O5 presented a wide range of heavy metal resistance, such as Pb, Cu, Cd, Hg, and Zn. In the study, we employed a system analysis strategy by integrating biochemical and comparative metabolomics to shed light on the adaptive mechanism of the polar bacterium to Cu^2+^. Our results indicated that strain O5 exhibited a relatively broad tolerance to Cu^2+^, Hg^2+^ Zn^2+^, Cd^2+^, and Pb^2+^, especially Pb^2+^ and Cu^2+^. Under copper pressure, strain O5 maintained intracellular redox balance by increasing antioxidant enzymes (SOD, GR) and antioxidant substances (GSH, Carotenoid). *Planococcus* sp. O5 in the presence of Cu stress achieved inherently different metabolite profiles, including amino acids, organic acids, and fatty acids. The adaption mechanism of strain O5 has been introduced in protein synthesis and repair, organic osmolyte accumulation, energy metabolism up-regulation, and fatty acids formation. This study laid a theoretical basis for revealing the response of biochemical and metabolomic mechanisms in polar bacterium to heavy metals, while also providing a new perspective on the bioremediation of metal-polluted environments.

## Data Availability

Not applicable.

## Supplementary Materials

The following supporting information can be downloaded at: https://www.mdpi.com/article/10.3390/toxics10060302/s1, Table S1: The list of differential metabolites between copper exposed group and untreated group in the logarithmic phase; Table S2: The list of differential metabolites between copper exposed group and untreated group in the stable phase; Table S3: The P-value and impact factor of significant pathways in the logarithmic phase; Table S4: The P-value and impact factor of significant pathways in the stable phase; Figure S1: Permutation test chart of copper exposed group and untreated group in the stable phase; Figure S2: Permutation test chart of copper exposed group and untreated group in the logarithmic phase.
