# Supplementary material for: Biochemical and Metabolomic Responses of Antarctic Bacterium Planococcus sp. O5 Induced by Copper Ion
Source: Toxics. 2022 Jun 2;10(6):302. doi: 10.3390/toxics10060302 (PMC9230899; doi:10.3390/toxics10060302)
Supplement: Supplementary file 1 [file toxics-10-00302-s001.zip › toxics-1721437-supplementary.pdf]

Supplementary Information

Biochemical and Metabolomic Responses of Antarctic Bacterium *Planococcus* sp.O5 Induced by Copper Ion

Tables

Table S1. The list of differential metabolites between copper exposed group and untreated group in the logarithmic phase

Number	Metabolite name	Log10P	Log2FC
1	Dodecanoic acid,	2.0512	2.8318
2	Phosphoric acid	1.578	2.4666
3	Stearic acid,	1.7272	2.0262
4	Palmitic acid	2.3481	1.7955
5	Lactic acid	1.4238	1.6293
6	Pyruvic acid	1.4048	1.2849
7	Lysine	1.5753	1.2454
8	Tyrosine	1.6752	0.97566
9	Glycine	2.6493	0.96566
10	Proline	1.4021	-0.62607
11	Methyl12-methyltridecanoate	2.4571	-1.0374
12	Ethyl palmitate	3.6517	-1.0948
13	Methyl14-methylpentadecanoate	5.7034	-1.3423

Table S2. The list of differential metabolites between copper exposed group and untreated group in the stable phase

Number	Metabolite name	$Log_{10}P$	Log_2FC
1	Phosphoric acid	4.3169	0.936
2	Lactic aicd	2.3401	0.9251
3	Pyruvic acid	1.6033	0.67483
4	Lysine	1.5348	0.67128
5	Glycine	1.6373	0.63144

Table S3. The P-value and impact factor of significant pathways in the logarithmic phase

Number	Pathway	P	-log(p)	Impact
1	Phenylalanine, tyrosine and tryptophan biosynthesis	0.0062527	2.2039	0.5
2	Glycine, serine and threonine metabolism	0.0027883	2.5547	0.24577
3	Pyruvate metabolism	0.0058839	2.2303	0.20684
4	Tyrosine metabolism	0.0038958	2.4094	0.13972
5	Glyoxylate and dicarboxylate metabolism	0.10377	0.98393	0.10582
6	Glycolysis / Gluconeogenesis	0.008253	2.0834	0.10044
7	Glutathione metabolism	0.075082	1.1245	0.08873
8	Arginine and proline metabolism	0.16561	0.78091	0.0778
9	Citrate cycle (TCA cycle)	0.00052271	3.2817	0.04634
10	Fatty acid biosynthesis	0.038151	1.4185	0.01473
11	Primary bile acid biosynthesis	0.093099	1.0311	0.00758

Table S4. The P-value and impact factor of significant pathways in the stable phase

Number	Pathway	P	-log(p)	Impact
1	Glycine, serine and threonine metabolism	0.0062527	2.2039	0.24577
2	Pyruvate metabolism	0.0027883	2.5547	0.20684
3	Glyoxylate and dicarboxylate metabolism	0.0058839	2.2303	0.10582
4	Glycolysis / Gluconeogenesis	0.0038958	2.4094	0.10044
5	Glutathione metabolism	0.10377	0.98393	0.08873

6	Arginine and proline metabolism	0.008253	2.0834	0.0778
7	Citrate cycle (TCA cycle)	0.075082	1.1245	0.04634
8	Primary bile acid biosynthesis	0.16561	0.78091	0.00758

Figures

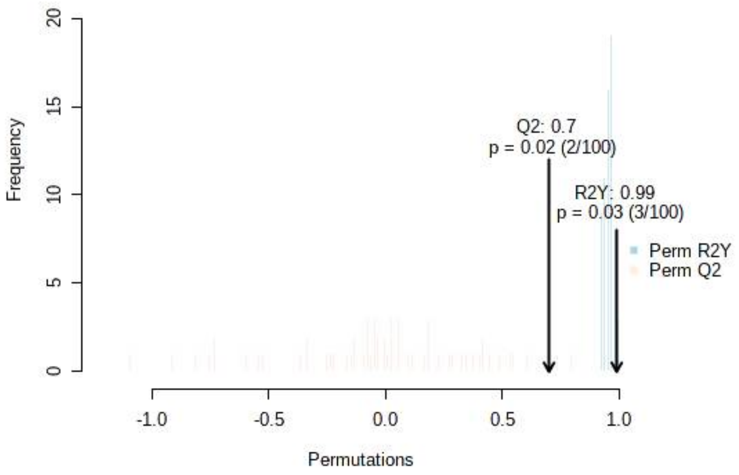

Figure S1. Permutation test chart of copper exposed group and untreated group in the stable phase

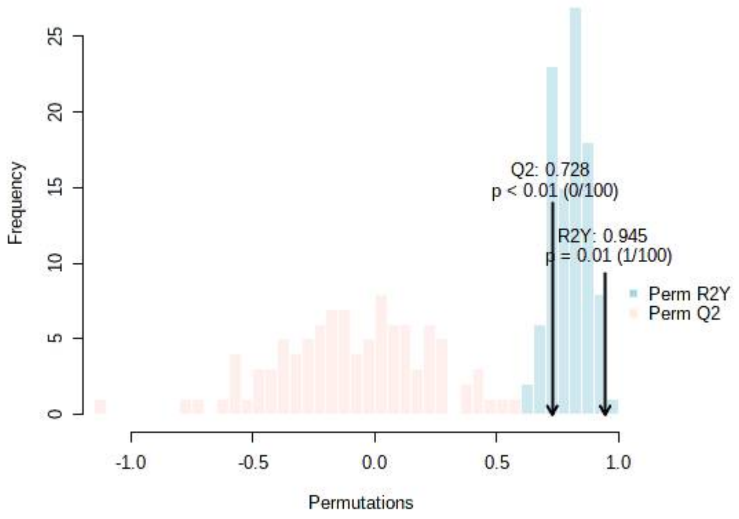

Figure S2. Permutation test chart of copper exposed group and untreated group in the logarithmic phase
